# Age-related serum vitamin B12 concentrations in epileptic patients receiving valproate monotherapy

**Published:** 2013

**Authors:** Mahmood Motamedi, Abdorreza Naser Moghadasi, Sanaz Ahmadi Karvigh

**Affiliations:** 1 Professor, Department of Neurology, Sina Hospital, Tehran University of Medical Sciences, Tehran, Iran; 2 Researcher, Sina Multiple Sclerosis Research Center, Sina Hospital, Tehran University of Medical Sciences, Tehran, Iran; 3 Neurologist, Sina Multiple Sclerosis Research Center, Sina Hospital, Tehran University of Medical Sciences, Tehran, Iran

**Keywords:** Age, Vitamin B12, Valproate

Long-term usage of antiepileptic drugs (AED) may lead to changes in serum vitamin B12 (B12) levels. Studies show that after sodium valproate prescription in epileptic patients, B12 concentrations increased.^1^ On the other hand, in the normal population, serum levels of B12 decreases with age.^2^

In the case of B12 deficiency, various hematologic, neurologic, and psychiatric clinical findings may be developed.^3^

Since there are few reports on the increasing of B12 levels in the patients using sodium valproate, there are some questions that have to be answered.

To evaluate the effects of sodium valproate monotherapy on serum B12 concentration of different age-groups we conducted the current study.

59 eligible patients (30 men and 29 women) were selected from the outpatient epilepsy clinic of Sina Hospital between September 2008 and September 2009. The inclusion criterion for patients was to have received sodium valproate monotherapy for at least one year. The exclusion criteria were any change in drug dosage during last year, receiving drugs with B12 component in the previous 2 months, and being a vegetarian. Moreover, the patients who had any concomitant medical disorders were also excluded from our study.

Their mean age was 25.7 ± 12.9 years, and the age range was 9 to 74 years. Mean duration of patients’ disease was 7.4 ± 3.3 years. Sodium valproate was used as monotherapy in all patients with average treatment duration of 3.7 ± 1.2 years.

Mean dosage of prescribed sodium valproate was 636.27 ± 269.8 mg/day. Statistical analysis showed that there was no significant difference between males and females in these characteristics (P > 0.05). Mean B12 level was determined as 524.24 ± 271.24 pg/ml in all patients. Furthermore, analysis of the correlation between the serum B12 and age showed a significant association, (r = -0.31, P = 0.01). This means that, in these patients, increase in age is associated with decrease in B12 levels. Our study showed that the B12 levels have a reverse relationship with the age of patients who received sodium valproate monotherapy.

Previous studies have shown that B12 serum concentrations decrease with age.^2^ While this decrease has been shown in different studies, there is no recommendation for the aged population to use B12 supplement.

Complications of B12 deficiencies have been discussed before.^3^ Sodium valproate could increase homocysteine and this rise is considered as a predisposing factor for cerebrovascular disease, dementia, and cognitive impairment.^4, 5^ On the other hand, lack of B12 increases homocysteine.^5^ All these findings can be concluded in a recommendation to use B12 supplements in elderly epileptic patients receiving sodium valproate monotherapy.

## References

[1] Gidal BE, Tamura T, Hammer A, Vuong A (2005). Blood homocysteine, folate and vitamin B-12 concentrations in patients with epilepsy receiving lamotrigine or sodium valproate for initial monotherapy. Epilepsy Res.

[2] Wolters M, Strohle A, Hahn A (2004). Age-associated changes in the metabolism of vitamin B (12) and folic acid: prevalence, aetiopathogenesis and pathophysiological consequences. Z Gerontol Geriatr.

[3] Oh R, Brown DL (2003). Vitamin B12 deficiency. Am Fam Physician.

[4] Karabiber H, Sonmezgoz E, Ozerol E, Yakinci C, Otlu B, Yologlu S (2003). Effects of valproate and carbamazepine on serum levels of homocysteine, vitamin B12, and folic acid. Brain Dev.

[5] Dorszewska J, Winczewska-Wiktor A, Sniezawska A, Kaczmarek I, Steinborn B (2009). Homocysteine and asymmetric dimethylarginine (ADMA) in epilepsy. Przegl Lek.

